# Issues of Feeding Strategy for Lactating Cows in Vietnamese Smallholder Dairy Farms

**DOI:** 10.3390/ani11030729

**Published:** 2021-03-08

**Authors:** Nguyen N. Bang, Nguyen V. Chanh, Nguyen X. Trach, Duong N. Khang, Ben J. Hayes, John B. Gaughan, Russell E. Lyons, Nguyen T. Hai, David M. McNeill

**Affiliations:** 1 School of Veterinary Science, The University of Queensland, Gatton, QLD 4343, Australia; dr.russ.lyons@gmail.com; 2 Faculty of Animal Science, Vietnam National University of Agriculture, Hanoi 131000, Vietnam; nxtrach@vnua.edu.vn; 3 Faculty of Animal Science and Veterinary Medicine, Nong Lam University, Ho Chi Minh 71308, Vietnam; chanh.nguyenvan@hcmuaf.edu.vn (N.V.C.); duongnguyenkhang@gmail.com (D.N.K.); hai.nguyenthanh@hcmuaf.edu.vn (N.T.H.); 4 Queensland Alliance for Agriculture and Food Innovation, The University of Queensland, St Lucia, QLD 4067, Australia; b.hayes@uq.edu.au; 5 School of Agriculture and Food Sciences, The University of Queensland, Gatton, QLD 4343, Australia; j.gaughan@uq.edu.au

**Keywords:** Napier grass, rice straw, milk production, feed efficiency, methane emissions, dietary imbalance, hierarchical clustering, complete pellets

## Abstract

**Simple Summary:**

Milk productivity of Vietnamese smallholder dairy cows is reported to be relatively low. Thorough analysis of lactating cow diets and feeding regimes in those smallholder dairy farms could help define the limitations of diets and feeding regimes relative to milk production. This study analysed and compared the feeding regimes and nutrient balance for lactating cows among four typical dairy regions including both highlands and lowlands located in both the north and south of Vietnam and evaluated the possibility of systematic dietary imbalance. The results show that the diets in all regions were excessive in protein, fibre and most mineral concentrations but insufficient in energy and non-fibre carbohydrates. The most used roughages including Napier grass, corn silage, fresh corn with cob, and rice straw were all relatively high in fibre concentrations. Feed efficiency of the diets across regions were sub-optimal. Thus, increasing dietary net energy concentration by increasing the use of starch and fat and decreasing the fibre concentration of the diet by decreasing the use of Napier grass or rice straw to balance the diets might help improve the milk production and thereby increase feed efficiency.

**Abstract:**

A limited literature suggests relatively simple feeding regimes and diet formulation strategies for dairy cows in Vietnamese smallholder dairy farms (SDFs). This study aimed to classify and compare feeding regimes and nutrient balance for lactating cows between four typical dairy regions (south lowland, south highland, north lowland, and north highland) in Vietnam and evaluate the possibility of systematic dietary imbalance. Eight SDFs from each of the four regions were visited for two adjacent milking periods per farm. For each visit, frequency and methods of feed and water supply to the lactating cows were recorded, and individual fat corrected milk yield (ECM) of lactating cows were calculated from milk yield and fat concentration. The amount of each diet ingredient offered and refused by each lactating group was weighed and sampled for calculation of dry matter intake per cow (DMI) and analysis of nutrient composition in the component offered. PCDairy, a diet formulation computer model, was used to calculate actual and recommended dietary nutrient concentrations and predict potential milk production. Factor analysis, cluster analysis, and ANOVA were applied to determine grouping effects across as well as between regions. Feeding regimes and diets were grouped into three and nine clusters, respectively. Farmers in the same region tended to apply similar diets and feeding regimes. Across regions, only 47% of all SDFs supplied water ad libitum to the cows. The most used roughages including Napier grass, corn silage, fresh corn with cob, and rice straw were all relatively high in neutral detergent fibre (NDF), acid detergent fibre (ADF), and acid detergent lignin (ADL). The diets in all regions were excessive in crude protein, NDF, ADF, ADL, and most minerals (Ca, P, Mg, K, Na, S, Fe, Zn, Cu, and Mn) but insufficient in net energy and non-fibre carbohydrate. Feed efficiency (1.06 kg FCM/kg DMI) of the diets were sub-optimal. Feeding regimes and dietary nutrient balance of the south lowland SDFs were most problematic. Increasing dietary net energy concentration by increasing the use of starch and fat and decreasing dietary fibre concentration by decreasing the use of Napier grass or rice straw to balance the diets might help improve the milk production and thereby increase feed efficiency.

## 1. Introduction

Although both commercial and SDF systems co-exist in Vietnam [1], Vietnam remains heavily reliant on smallholder dairy farms (SDFs) for its domestic supply of fresh milk. There were approximately 28,695 SDFs in 2016 [2,3], producing more than 80% of Vietnam’s fresh milk [4]. However, average daily milk yields of these SDF cows, despite recent improvements, remains relatively low at 14–15 kg/cow/day in southern [5] and northern [6,7] provinces. This not only impacts national milk production but also the milk production efficiency of SDFs. Although the reasons for low productivity can be multifactorial, poor nutrition is usually considered a most likely reason [8]. High genetic merit Holstein cows, such as those being increasingly imported into Vietnam, can only support high milk yield if their nutritional requirements are met [9]. 

Thorough analysis of lactating cow diets in the SDFs, relative to requirements, is required to define dietary limitations. To do that, the dietary ingredients and amounts of each that SDF farmers offer the cows and nutrient compositions of each must be determined. From that the dietary supply of key nutrients or nutrient groupings (crude protein, CP; neutral detergent fibre, NDF; acid detergent fibre, ADF; acid detergent lignin, ADL; non-fibre carbohydrate, NFC; Starch; and key minerals, such as Ca and P) can be calculated. Similarly, requirements can be calculated once key information on the cow is available (body weight, lactation number, days in milk, milk yield, milk fat and protein concentrations, targeted body weight gain, days of pregnancy). From these data, the requirements can be estimated by using the mathematical nutritional guidelines of National Research Council (NRC, America), Agricultural Research Council (ARC, United Kingdom), National Institute for Agricultural Research (INRA, France), or Commonwealth Scientific and Industrial Research Organisation (CSIRO, Australia) [5,6].

Although these calculation can be done manually, many computer-based nutrition models such as PCDairy, Cornell Net Carbohydrate and Protein System, CamDairy, Feed into Milk, Molly, and Rumen8 [10,11,12] are now available to assist. However, in the situation of Vietnamese SDFs, there appears to have been no systematic published work on the required inputs for such models.

Only a few surveys have mentioned examples of locally relevant input data required for modelling dietary diets on SDF diets in Vietnam [8,13,14,15,16]. Fresh Napier grass (*Pennisetum purpureum*) appears to be the main type of forage used; followed by agricultural by-products such as rice straw, corn stalks (after the grain is removed), banana stalks; then other cultivated tropical grasses such as Ruzi grass (*Brachiaria ruziziensis*), Guinea grass (*Panicum maximum*), Signal grass (*Brachiaria decumbens*), Long Tay grass (*Brachiaria mutica*), and Mulato (*Brachiaria ruziziensis* × *Brachiaria decumbens* × *Brachiaria brizantha*); and finally some naturally available forages collected from river banks or fallow areas [13,14]. Commercial concentrate pellets, often called “complete pellets” in Vietnam and commonly purchased from milk processing companies or feed companies linked to the milk companies, are the main type of concentrate fed. SDF farmers appear to not commonly mix their own concentrates or mix concentrates into the forage components; they are mainly fed separately to the forage. In Ho Chi Minh City, a city in the south of Vietnam, Lam et al. [15] reported lactating cow diets of 48.6% (dry matter basis) roughage such as Napier grass, Guinea grass or rice straw; 19.5% by-products such as brewers’ grains; and 31.9% concentrates. Cows were fed twice a day and depending on the milk yield of the cows and the availability of the roughage, they were fed (fresh basis) about 20–40 kg of roughage and 4–6 kg of commercial concentrate per day [15,16]. Concentrate was fed before milking and roughage after [15]. In Son La, a highland province in the north, Cuong et al. [8] reported that cows were fed diets comprising 51.8% roughage and 48.2% concentrate. Napier grass and Signal grass were fed ad libitum and commercial concentrates were fed at a ratio of 0.5 kg concentrate per 1 kg of milk [8]. Roughage was fed to cows first, and then concentrate was fed to cows during milking [8]. Although the information from these studies was insufficient to allow the reliable analysis of nutrient concentrations in the diets, it suggests potential for diets to be regionally specific and imbalanced. The tropical roughages most commonly used are considered to be usually low in CP, high in NDF and ADF, and low in dry matter digestibility and therefore net energy concentration [17,18]. Similarly, the simple rules used to add roughage to concentrate indicate the risk of dietary imbalance and health concerns such as ruminal acidosis.

Realising the lack of published scientific analysis of feeding regimes and diets for lactating cows in Vietnamese SDFs, this study was conducted to classify and compare the feeding regimes and diets for lactating cow in four typical but geographically contrasting dairying regions of Vietnam and define the likely dietary imbalances within and across these regions. It was hypothesised that the feeding regimes and lactating cow diets are specific to each region; and across regions, the lactating cow diets are excessive in fibre, with insufficient nutrient concentration for high yielding cows. The overall aim was to identify limitations of current feeding strategies for lactating cows across regions and suggest possible nutritional interventions.

## 2. Materials and Methods

### 2.1. Farm Selection and Farm Visits 

This study was conducted from 24 August to 7 October 2017 on 32 SDFs which were located in four typical dairy regions of Vietnam including a south lowland region (SL) (Cu Chi district, Ho Chi Minh, Vietnam), a south highland region (SH) (Don Duong district, Lam Dong, Vietnam), a north lowland region (NL) (Ly Nhan and Duy Tien districts, Ha Nam, Vietnam), and a north highland region (NH) (Moc Chau district, Son La, Vietnam) with eight farms per region. These four regions were chosen as they are the representatives of the main SDF regions of Vietnam with high dairy cow populations, located in both lowlands and highlands in both the north and the south, and with both long dairy farming history (SL, SH, and NH) and short dairy farming history (NL) [19]. Some central provinces such as Thanh Hoa, Nghe An, and Ha Tinh also have a high number of dairy cows, but these were not chosen as the dairy farms in those regions are mainly large commercial farms owned by milk-processing companies [19].

The eight SDFs per region were selected randomly from 40 SDFs per region that had previously been included in a collaborative economic survey of SDFs [20,21]. In that economic survey, 40 SDFs per region were randomly chosen from the lists of SDFs supplied by the regional District Agriculture Departments. In the current study, eight SDFs per region were randomly selected from the SDFs in that economic survey who agreed to continued involvement in further studies. The selected SDFs were contacted by phone to inform their managers of the purpose and content of the study and to confirm their involvement. 

This study was conducted concurrently with other studies by the same authors and on the same SDFs [22,23,24]. Each SDF was visited for an afternoon and the next morning either side of and during milk times when feeds were being offered and milk collected to allow measurement of the inputs necessary to use the PCDairy model to calculate predicted against actual milk yield.

### 2.2. Feeding Regime

A trained team of 3–4 observers visually recorded feeding regime per farm. The “Feeding regime dataset” included eight qualitative and four quantitative variables (Table 1). In Table 1, two feeding times were counted as two if one feeding occurred at least 3 h from the other. Partial mixed ration was a mixture of corn silage, concentrates, and minerals which farmers bought from feed processing companies. Quantity of water supplied was classified as ad libitum when water trough was always full and accessible for the cows, moderately when water trough was not always full or full but too small for the whole herds, and insufficient when water trough was empty for an hour or more and the cows were observed waiting near the water trough. Quality of water supplied was classified as good when water was clear with no smell, medium when water was clouded with visual contaminations but has no strange colours or smells, and poor when water was clouded and has moss green colour, yellow colour, dark colours, mouldy odour, or fermented odour.

### 2.3. Diets

#### 2.3.1. Feed Intake “As Fed”

At feeding times, each type of feed offered to lactating herd was weighed using a digital hanging scale Model OCS M 100 (Vietnam Japan Digital Scale Company, Ho Chi Minh city, Vietnam) [25] (Figure S1a–c). In all farms, concentrates were eaten completely and only a small amount of roughage (<3% offered) was leftover. The refusal roughages were collected, separated, and weighed. Intake “as fed” (kg/day) of each feed type by the whole lactating herd was calculated as total offered amounts minus refusal amount of that feed. The intake “as fed” of each feed type by a cow was calculated as the total intake “as fed” of that feed by the whole lactating herd divided by total number of cows consuming that feed. 

#### 2.3.2. Nutrient Composition Analysis

Approximately 1 kg each type of roughage and 400 g each type of concentrate used by each SDF were sampled and stored in 30 cm × 30 cm plastic sealable bags and frozen in a fridge. After all feed samples had been collected, the same feed types samples within each region were counted. For the feed types with more than two samples per region, the samples were mixed, with the same weight ratio in the mix per initial sample, and a new sample taken for a nutritional panel analysis. There were two exemptions to this rule. There were more samples of corn powder per region, but the nutrient composition of this feed was available and sufficient, thus they were not analysed. In contrast, there was only one sample of passion fruit pulp, but nutrient composition of this feed was not available, thus it was analysed.

The samples were dried to determine dry matter concentration (DM) and to be transferred to dry samples at Animal Nutrition Laboratory, Faculty of Animal Science, Vietnam National University of Agriculture. After that, the dry feed samples were ground and sent to Dairy One Forage Laboratory, Ithaca, NY, USA for analysis of other chemical components by traditional wet chemistry methods described in details at Laboratory Analytical Procedures [26,27]. Feeds were analysed for the following chemical compositions: net energy for lactation (NEL), CP, NDF, ADF, ADL, fat, NFC, starch, total digestible nutrients (TDN), total ash (Ash), calcium (Ca), phosphorus (P), magnesium (Mg), potassium (K), sodium (Na), sulphur (S), iron (Fe), zinc (Zn), copper (Cu), and manganese (Mn).

The chemical compositions of the feed types, which were used by farmers but were not analysed in the laboratory, were derived from available feed library of PCDairy [12] and available feed nutritive value books [18].

#### 2.3.3. Dry Matter Intake

From the intakes “as fed” of 19 feed types and the DM of each feed, the DM intake (DMI) of each feed was calculated per cow per farm, and this became the “Diet dataset”.

### 2.4. Identification of Dietary Imbalance

#### 2.4.1. PCDairy

PCDairy version 2015 (Figure S1d), a computer-based mechanistic nutrition model which was developed by the University of California Davis, was used. PCDairy estimates nutrients supplied by diets, from an inputted feed library, and nutrient requirements of animals based on the empirical nutrition models of NRC [9] and estimate methane emission from the diets based on the equations of Moraes et al. [12,28,29]. PCDairy was chosen as it has been made available, free for use by Vietnamese farmers, nutritionists, and extensionists through a cooperation program of the US Department of Agriculture, University of California Davis, and the Ministry of Agriculture and Rural Development (Vietnam) [30,31]. In addition, PCDairy was the only available nutrition model which has been translated into Vietnamese to make it as user friendly as possible.

#### 2.4.2. Background Data of Lactating Herds

PCDairy required the background data of lactating herds including number of cows, cow breed, average lactation, days in milk, milk yield (kg/cow/day), milk fat concentration (%), body weight (BW, kg), daily weigh gain, and level of energy adjustment for activity. Almost those background data were obtained from other studies that were conducted concurrently with this study by the same authors and on the same SDFs [22,23,24].

The background data of the lactating herds in each region are summarised in Table 2. Briefly, at each visit, the total number of lactating cows were counted, and the farmers were asked to check their recording and/or memory to provide breed, lactation number, and days in milk for each cow [22,23]. This information was used to calculate the percentage of the first and second lactation cows and average days in milk of each herd. Dimensions of the cowsheds were measured to calculate the space allowance per cow [24].

Morning and afternoon milk yields were weighed per cow and summed to obtain single-day milk yield [22]. Morning and afternoon milk were sampled per cow for analysis of milk fat concentration by gravimetric method at the Nutrition Laboratory, Vietnam National University of Agriculture [22]. Single-day milk yield was converted to fat corrected milk (FCM, 3.5% fat) so that the actual FCM production can be comparable with the diet-allowable FCM (3.5% fat) predicted by PCDairy software. Specifically, FCM (3.5% fat) was calculated using the equation of Britt et al. [32]:

FCM (kg/cow/day) = 0.432 × milk yield (kg/cow/day) + 16.23 × fat yield (kg/cow/day).

Body weight (BW, kg) of each cow was estimated from heart girth using an equation suggested for cattle by Goopy et al. [33]. The heart girth of each lactating cow was measured by draping the tape around the girth closest to the heart [34].

Daily weight gain was set up as the default value of 0 kg weight gain per day [12]. Level of energy adjustment for activity was set up as 5% of energy requirement for maintenance because the cows in the current study had limited space allowance to move inside the cowsheds [12,24].

#### 2.4.3. Analysing Lactating Cow Diets by PCDairy

When using PCDairy, firstly, the FEEDLIST package was used to update the feed library with the price of all feed and nutrient profile of new feeds. The prices of the feed types were obtained by asking the farmers and averaged per feed type. Then, the ANLSIS-L package was used to analyse the diets. 

For each SDF, ANLSIS-L was used for four tasks which require slightly different input data. Task 1 was calculating dry matter intake, cost, and actual nutrient concentrations of the diets fed to cows. Task 2 was modelling the recommended nutrient concentrations for production of observed FCM (called PCDairy Recommendation 1). Task 3 was modelling the recommended nutrient concentrations for the production of a target FCM (3.5% fat) yield of 25 kg/cow/day (PCDairy Recommendation 2). Finally, Task 4 was predicting NEL-allowable FCM, CP-allowable FCM, and methane (CH_4_) emission potential per unit of FCM from the cows given a diet. For all tasks, the data including intakes “as fed” of dietary ingredients, percentage of the first and second lactation cows in each lactating herd, average days in milk, BW, daily body weight change, and NEL added for activity were entered into ANLSIS-L package. The different input data ANLSIS-L between Tasks 1, 2, and 4 with Task 3 was that the actual milk yield and milk fat concentration were entered for Task 1, 2, and 4, while a target milk yield of 25 kg FCM/cow/day and milk fat of 3.5% was entered for Task 3.

By comparing the actual nutrient concentrations with PCDairy Recommendation 1, the particular nutrient that deficient in the actual diet for the production of the actual FCM can be determined [12]. Similarly, by comparing the actual nutrient concentrations with PCDairy Recommendation 2, the particular nutrient that deficient in the actual diet for the production of a target FCM of 25 kg/cow/day can be determined [12]. In addition, the balance between dietary NEL and CP of the actual diets can be determined by comparing NEL-allowable FCM and CP-allowable FCM. 

### 2.5. Statistical Analysis

All statistics were performed using the base and additional packages of R software [35].

#### 2.5.1. Statistical Comparisons

Farms were the experimental unit in all analyses. Descriptive statistics for quantitative variables were calculated for each region using the “psych” R package [36]. The normality of quantitative variables was tested using both the Shapiro–Wilk test and histograms. Kruskal–Wallis tests followed by Dunn post-hoc tests (*p* < 0.05), using the “FSA” R package [37], were applied to compare the medians of the not-normally distributed variables. One-way ANOVA tests followed by Tukey–Kramer tests (*p* < 0.05), using the “agricolae” R package [38], were applied to compare the means of the normally distributed variables. Frequencies of each sublevel of qualitative variables were compared by Fisher’s exact tests followed by Bonferroni-corrected pairwise Fisher’s exact tests (*p* < 0.05), using R “rcompanion” package [39].

#### 2.5.2. Multivariate Statistical Analysis

Hierarchical Clustering on Principal Components (HCPC) analysis method was applied to cluster Feeding regime dataset (12 variables) and Dietary dataset (19 variables), which include many inter-correlated variables [40,41]. Based on HCPC analysis, three standard methods including principal component methods, hierarchical clustering method, and partitioning clustering method were applied to cluster the farms into the groups so that the farms in the same group are more similar to each other than to those in other groups [42,43]. Firstly, depending on the type of the dataset, either Principal Component Analysis method or Factor Analysis of Mixed Data method was applied to transform the dataset into non-correlated principal components. In the current study, Principal Component Analysis was applied to the Diet dataset as this dataset included only quantitative variables while Factor Analysis was applied to Feeding regime dataset as this dataset included both quantitative and qualitative variables. After that, to reduce noise and increase cluster stability in the data, hierarchical cluster analysis was applied on only some first principal components to identify an initial number of clusters. The decision of how many and which principal components to keep was made based on Kaiser’s criterion; all principal components with an eigenvalue ≥ 1.00 were initially retained [44]. Additionally, the cumulative percentage of variance explained by the retained principal components was cross-checked to make sure it was ≥70% [40]. Finally, the k-means clustering method was applied to identify an optimum number of clusters and assign farms into each cluster [42].

To further characterise each cluster in the final sets of clusters, V-tests [45] were used. For quantitative variables, V-tests compared the mean of each variable in each cluster to the mean of that variable in the whole the dataset [40]. For qualitative variables, V-tests compare the percentage of each category of each qualitative in each cluster to the percentage of that category in the whole the dataset [40].

All the multivariate statistical analyses were performed using R package “FactoMineR” [46] and visualised using R package “factoextra” [47]. The results of the HCPC analysis were visualised as dendrograms.

## 3. Results

### 3.1. Feeding Regime

The feeding regime dataset is presented, per region, in Table 3. In NL, both concentrates (four times per day) and roughages (four times per day) were offered more frequently than in all other regions, with the exception of SL where roughages were offered at a similar level to NL. Across regions, concentrates were offered on average two times per day and roughages three times per day. Feed troughs were cleaned approximately twice as often in NL compared to NH and SH, with SL in between (*p* < 0.05). Across regions, feed troughs were cleaned an average of 10 times per week.

Only 15 out of all 32 SDFs supplied water ad libitum to the cows and the majority of these SDFs were in NL (7 SDFs) and NH (6 SDFs) (Table 3). All SDFs in SL only supplied water to the cows after feeding concentrate. None of the SDFs in SL supplied water ad libitum to the cows. Similarly, only two out of eight SDFs in SH supplied water ad libitum for the cows. 

None of SDFs took the dry matter of feeds into account when determining amounts to offer, and only three out of 32 SDFs across regions weighed feed ingredients before feeding (Table 3). None of the SDFs mixed concentrates and roughages prior to feeding time. One SDF in SH and four in NL mixed concentrates with roughages during feeding time. All SDFs in SL and all but one in NH fed concentrates separately to roughages. 

**Figure 1:**
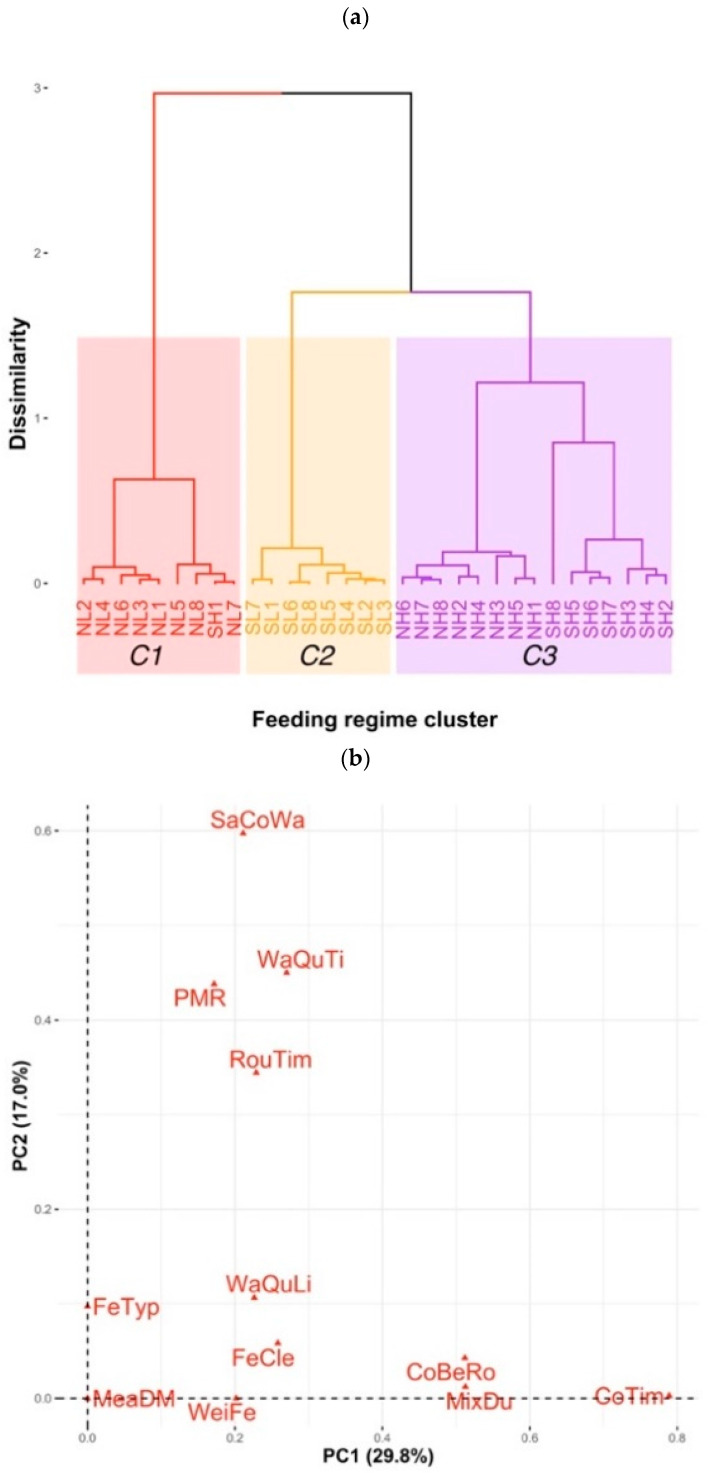

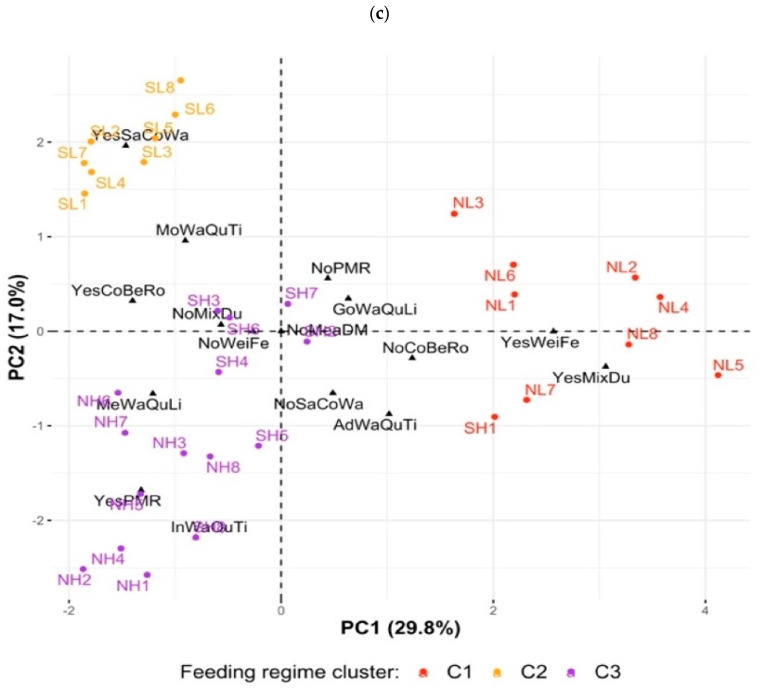
Results of Factor Analysis and Hierarchical Clustering on Principal Components (HCPC) for feeding regime data. SL1-SL8, SH1-SH8, NL1-NL8, amd NH1-NH8 represent the feeding regimes in eight farms in south lowland, south highland, north lowland, and north highland, respectively. Feeding regime variables: FeTyp, types of feed used; FeTim, times of feeding roughages per day; CoTim, times of feeding concentrates per day; FeCle, times of cleaning roughage trough per week; WaQuTi, if water was supplied ad libitum (AdWaQuTi), moderately (MoWaQuTi), or insufficiently (InWaQuTi); WaQuLi, if water quality was medium (MeWaQuLi) or good (GoWaQuLi); SaCoWa, if the same trough was used for both concentrate and water (YesSaCoWa) or not (NoSaCoWa); WeiFe, if feeds were weighed when feeding (YesWeiFe) or not (NoWeiFe); MeaDM, if farmers measured feed dry matter (YesMeaDM) or not (NoMeaDM); PMR, if partial mixed ration was used (YesPMR) or not (NoPMR); CoBeRo, if concentrates were fed before roughage (YesCoBeRo) or not (NoCoBeRo); MixDu, if concentrates and roughages were mixed during feeding time (YesMixDu) or not (NoMixDu). PC1 and PC2, Principal Components 1 and 2. (**a**) HCPC, cluster dendrogram depicting the three optimum feeding regime clusters of C1–C3; (**b**) first two PC view of all variables; and (**c**) first two PC view of qualitative variables and observations.

From the Feeding regime data on the 13 variables (Table 3), the FAMD analysis defined the first five principal components accounting for 78.0% of the total variance. HCPC, based on those first five principal components, defined three optimum feeding regime clusters (Figure 1a). All NL SDFs and one SH SDFs grouped into the feeding regime Cluster 1 (coloured in red), all SL SDFs in Cluster 2 (yellow) and all NH and other SH SDFs in Cluster 3 (purple).

The directionality and the amount of variation of feeding regime variables and the associations of these variables with the feeding regime clusters are presented in a two-dimensional view of the first two principal components (Figure 1b for all variables and Figure 1c for sublevels of qualitative variables and feeding regime clusters). The qualitative variables that varied most (furthest from the original coordinates in Figure 1b,c) and were most meaningful in the partitions of the clusters were “using the same trough for water and concentrate (SaCoWa), yes or no”, “concentrate feeding times (CoTim)”, “water quantity (WaQuTi), insufficient, moderate, or ad libitum”, “mixing concentrate and roughage during feeding (MixDu), yes or no”, and “using partial mixed ration (PMR), yes or no”.

The main characteristics of each feeding regime cluster are presented in Figure 1c and Table S1. Cluster 1 (all NL SDFs in red) was characterised by mixing feed during feeding and weighing feed before feeding (red SDFs are close to sublevels “YesMixDu” and “YesWeiFe” in Figure 1c). However, Figure 1c was only the visualised relationships of feeding regime cluster and feeding regimes variables in the view of two first principal components. V-tests described better the main characteristics of each feeding regime cluster by statistically comparing the mean of each quantitative variable in each cluster with the mean of that quantitative variable in the whole dataset and comparing the percentage of categories of each qualitative variable in each cluster to the percentage of that category in the whole dataset. The results of V-tests (Table S1) show that the SDFs in Cluster 1 (all NL SDFs) fed cows concentrates and roughages more times per day and cleaning feed troughs more times per week than average. In addition, more SDFs in this cluster than average fed concentrates and roughages at the same time and mixed them during feeding, supplied cows ad libitum good quality water, did not use the same trough for both water and concentrates, and did not use partial mixed ration. SDFs in Cluster 2 (all SL SDFs) fed cows concentrates fewer times per days than average. In addition, more of the SDFs in this cluster than average used the same trough for both concentrates and water, fed cows concentrate before roughage, and supplied cows with moderate quality water than average, but fewer of the SDFs in this cluster supplied cows water ad libitum than average. SDFs in Cluster 3 (all NH and seven SH SDFs) fed concentrates and roughages fewer times per day than average, used partial mixed ration more, did not use the same trough for both water and concentrates, and did not mix concentrates and roughages during feeding than average.

### 3.2. Diets

The number of SDFs using a given feed type and the average DM intake/cow for each feed per region are summarised in Table 4. A similar number of feed types was offered per region (4–5 types, *p* = 0.845, Table 3). Nineteen feed types were used by SDFs across regions. Eight SDFs reported using sodium chloride either in the silage-making process or by spreading it over the feed at feeding time. Five SDFs reported occasionally using calcium supplements, two reported using mineral blocks, two reported using bypass fat, and one reported using sodium chloride, glucose, and whey. However, we either did not observed or could not measure the actual amounts of these feed additives, hence they were not included in the dietary calculations in this study.

The most popular roughage across regions was Napier grass (Table 4). Five to eight SDFs per region fed Napier grass and the average amount fed per cow was 3 kg DM/day. Next was corn silage. which was popularly used in NH (8 SDFs), NL (7 SDFs). and SH (4 SDFs) but not at all in SL. Cows in NH were fed the most corn silage (5 kg DM/day, *p* < 0.001) and the least Napier grass (1.2 kg DM/day, *p* = 0.009). Dry rice straw was used the most in SL (five out of eight SDFs) but not at all in any of the other regions. Fresh tropical grasses other than Napier were used by 1–2 SDFs in SL, NL, and NH. Fresh corn with cob was used by 1–2 SDFs in SL, SH, and NH.

Concentrate pellets were the main concentrate source for cows in all SDFs in all regions (6.6 kg DM/cow/day, *p* = 0.112, Table 4). When the mean amount of concentrate used across region was divided by mean FCM production across regions (16.9 kg FCM/cow/day), the ratios was 0.39 kg of concentrate pellets per kg of FCM. The next most popularly used concentrate was brewers’ grain, which was used in SL, SH, and NH but not at all in NL. Corn powder and whole roasted soybean meals were used at a moderate to low extent only in SH and NL. Partial mixed ration was only used in NH and was used by all SDFs in this region. Other feeds such as passion fruit pulp, sweet potato tuber, dried distillers’ grain, and rice grain with husk were used by 1–2 SDFs, in SH only.

**Figure 2:**
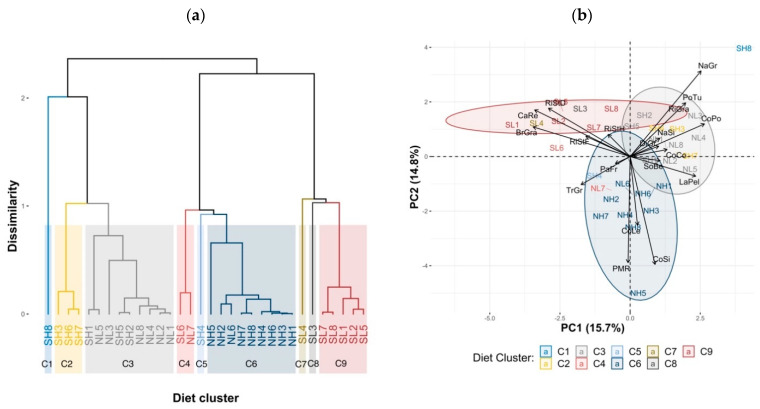
Results of Principal Component Analysis and Hierarchical Clustering on Principal Components (HCPC) for diet data. SL1-SL8, SH1-SH8, NL1-NL8, and NH1-NH8 represent the cow diets in the farms numbered 1-8 in south lowland, south highland, north lowland, and north highland, respectively. Nineteen feed intake variables (kg DM/day) were: NaGr, Fresh Napier grass; TrGr, Fresh tropical grass; CoCo, Fresh corn with cobs; CoLe, Fresh corn leaves; CoSi, Corn silage; NaSi, Napier grass silage; RiStF, Fresh rice straw; RiStD, Dry rice straw; RiStH, Rice hay; PMR, Partial mixed ration; LaPel, concentrate pellets for lactating cows; BrGra, Brewers’ grain; CaRe, Cassava residue; CoPo, Corn powder; SoBe, Whole soybean meal; PaFr, Passion fruit pulp; PoTu, Sweet potato tuber; DiGr, Dried distillers’ grain; RiGra, Rice grain with husk. PC1 and PC2, Principal Components 1 and 2. (**a**) Cluster dendrogram depicting the nine optimum diet clusters of C1–C9; and (**b**) biplot drawn in a two-dimensional view of the first two PCs.

From the Diet data on the on the 19 feed types (Table 4), the FAMD analysis defined the first nine principal components accounting for 79.9% of the total variance. HCPC, based on those first nine principal components, defined nine optimum diet clusters (Figure 2a). Similar to Feeding regime data, almost diets in the same region clustered into the same group except diets in the farms SH8, SL3, SL4, SL6, and NL7 which separated, mainly individually, from the main clusters. The largest clusters were Cluster 3 (six NL and three NH SDFs coloured grey), Cluster 6 (eight NH and one NL SDFs in dark blue), and Cluster 9 (five SL SDFs in dark red).

The directionality and the amount of variation of feed intake variables and the associations of these feed intake variables with the diet clusters are presented in the biplot in a two-dimensional view of the first two principal components (Figure 2b). Visually, the variables that varied most (longest arrows) and were most meaningful in the partitions of the clusters were cassava residue, brewers’ grain, and dry rice straw (close to Cluster 9); fresh Napier grass and sweet potato tuber (close to Clusters 1 and 3); and corn silage and partial mixed ration (close to Cluster 6). 

Further characterisation of each diet cluster by V-test (Table S2) showed that rice grain and sweet potato tuber were used most in C1 (one SDF only, in SH); dried distillers’ grain and fresh corn with cobs in C2 (three SDFs only, all in SH); corn powder, fresh Napier grass, and whole soybean meal in C3 (mostly NL but some SH); fresh tropical grass in C4 (two SDFs only, SL and NL); passion fruit pulp in C5 (one SDF only, in SH); partial mixed ration and corn silage in C6 (all eight SDFs in NH and one in NL); fresh rice straw and cassava residue in C7 (one SDF only, in SL); rice hay in C8 (one SDF only in SL); and dry rice straw, cassava residue, and brewers’ grain in C9 (five SDFs, all in SL). Figure 2b shows these aspects in the first two principal components.

### 3.3. Nutrient Composition of Commonly Used Feeds

Among feed types, 11 feed types (24 samples across regions) commonly used for dairy cows in each region were analysed for nutrient composition and are presented in Table 5. Another eight feed types including Napier grass silage, fresh corn leaves, fresh rice straw, rice hay, corn powder, rice grain with husk, dried distillers’ grain, and sweet potato tuber were not analysed. The nutrient compositions of those feeds were obtained from the literature [12,18] due to the very low content of those feeds in the diets (Table 4) and are summarised in Table A1, Appendix A.

Nutrient compositions (DM as per cent of fed and other nutrients on DM basis) of fresh Napier grass, corn silage, fresh corn with cob, brewers’ grain, and whole soybean meal varied widely across regions (Table 5). For example, the DM, NEL, CP, ADF, fat, starch, and ADL concentrations of Napier grass, the starch concentration of fresh corn with cob, the ADL concentration of brewers’ grain, and the fat concentration of whole soybean meal varied widely across regions using those roughages. Unlike roughage, the main nutrient concentrations of concentrate pellets varied slightly across regions.

Comparing roughages, as expected, concentrations of NEL (0.68 Mcal/kg), CP (7.2%), and NFC (6.8%) of dry rice straw were lowest among the analysed roughages while concentrations of NDF (77.6%) and ADF (50.5%) of this feed were highest among analysed roughages. In addition, as expected, DM concentrations of corn silage (23.8–26.3%), tropical grass (20.8–21.6%), and fresh corn with cob (24.4–25.0%) were higher than the DM concentration of Napier grass. However, not as expected, concentrations of NEL, CP, fat, NFC, NDF, ADF, ADL, and minerals of corn silage samples in NL and NH were all within the ranges of corresponding nutrients of Napier grass. CP concentration of SH silage (10.4%) was also within the range of CP concentration in Napier grass. Only corn silage in SH had lower concentrations of NDF and ADF and higher concentrations of NEL, NFC, and starch than those of Napier grass. Concentrations of NEL, CP, fat, NFC, NDF, ADF, and ADL of tropical grass and fresh corn with cob were not much different from those of Napier grass. 

NDF, ADF, and ADL concentrations of all roughages were high. Across roughages ADL concentration across roughages ranged from 5.1% in fresh corn with cob to 11.5% in NH fresh Napier grass. NDF and ADF concentrations ranged from 37.4% and 56.3%, respectively, in SH corn silage to 50.5% and 77.6%, respectively, in dry rice straw.

### 3.4. Cow Intake and Nutrient Concentrations of the Diets

Calculated DMI and dietary nutrient concentrations for average diets per region compared to nutrient concentration targets recommended by PCDairy are summarised in Table 6. Although DMI was highest (17.3 kgDM/day) in NH, similar for SH and NL, and lowest in SL (14.4 kgDM/day) (*p* = 0.007), DMI calculated as per cent of cow BW was similar across regions (3.2% BW, *p* = 0.861). Dietary concentration of DM was highest in NH diets (39.2%) and lowest in SH diets (32.3%) (*p* = 0.002).

The dietary concentration of NEL was similar across regions, 1.40 MCal/kg (*p* = 0.176). However, this concentration was lower than the NEL concentration recommended by PCDairy for either observed FCM production or a target production of 25 kg of FCM (1.50 MCal/kg and 1.59 MCal/kg, respectively). The dietary concentration of CP was highest in NH (17.5% DM) and SL (17.1% DM), followed by SH (16.4% DM), and lowest in NL (14.8% DM) (*p* = 0.003). The dietary CP concentrations in all regions were higher than the CP concentration (15.7%) recommended by PCDairy for the target of 25 kg of FCM.

Mean concentrations of NDF (45.8% DM) and ADF (27.3% DM) in the diets were similar across regions (*p* > 0.192) and much higher than the lowest concentrations suggested by PCDairy, which were 28% DM and 21% DM for NDF and ADF, respectively. Dietary concentration of fat in all regions was higher than the recommended level of 3% DM. Dietary fat concentration was highest in NL (4.1% DM) and lowest in NH (3.4% DM) (*p* = 0.026). Mean concentrations of TDN (64.3% DM) and NFC (27.4% DM) in the diets were similar across regions (*p* > 0.05). Starch concentration was highest in SH (22.6% DM) and lowest in SL (16.7% DM) (*p* = 0.04) while ADL concentration was highest in NH (6.8% DM) and lowest in SH (5.5% DM) (*p* = 0.012).

Concentrations of all the measured minerals in all regions were higher than the recommended concentrations and lower than the maximum concentration recommended by PCDairy.

### 3.5. Efficiencies of the Diets

Milk production, diet cost, and diet efficiencies of regions are summarised in Table 7. NEL-allowable FCM was similar across regions (18.6 kg FCM/cow/day) while CP-allowable FCM was highest in NH (30.9 kg FCM/cow/day) and lowest in NL (23.9 kg FCM/cow/day) (*p* < 0.001). The ratios of CP-allowable FCM:NEL-allowable FCM were 1.4 in NL, 1.5 in SL and SH, and 1.6 in NH. Thus, the maximum predicted FCM in all regions was taken as NEL-allowable FCM. 

Means of actual FCM in SL, SH, and NL farms were 2.3, 4.4, and 0.7 kg/cow/day lower than the means of predicted FCM for those regions, while the mean of actual FCM in NH was 0.6 kg/cow/day higher than predicted. The correlation coefficient^®^ between predicted and actual FCM was 0.59 (*p* < 0.05).

The feed efficiencies (kg of FCM produced/kg of DMI) of the diets in SL and SH (0.99 and 1.00 kg of FCM/kg DMI, respectively) were both lower than that of diets in NH (1.19 kg of FCM/kg DMI) (*p* = 0.016). Mean of diet costs across regions was 6.4 USD/cow/day, of which the concentrate costs accounted for between 52.6% (in NH) and 77.8% (in SL) of the total cost. Diet cost, calculated as either USD/cow/day or USD/kg DMI, was lowest in SL and highest in NH (*p* < 0.001). However, the cost of milk production from the diet, calculated as USD/kg FCM, was similar across regions (0.39 USD/kg FCM, *p* = 0.846).

Both predicted methane emissions calculated as per cent of dietary gross energy and as g/cow/day were similar across regions (*p* > 0.049). Environmental efficiency of the diets calculated per unit of DMI was also similar across regions (20.1 ± 0.3 g CH_4_/kg DMI, *p* = 0.064). However, environmental efficiency of the diets calculated per unit of FCM was poorest in SL (21.4 g CH_4_/kg FCM) and best in NH (16.5 g CH_4_/kg FCM) (*p* = 0.013).

## 4. Discussion

As hypothesised, feeding regimes and lactating cow diets were specific to regions and SDFs across regions tended to apply the same feeding regimes and diets for cows. The quality of the main forages used across regions were low and the diets across regions were unbalanced, showing excess NDF, ADF, CP, and key minerals but insufficient NEL, NFC, and fat. Improving the quality of roughage and rebalancing the diets, particularly for net energy and fibre concentrations, should be promoted as key strategies for further research and practical assessment.

### 4.1. Feeding Regimes

There were only three feeding regime clusters. As the hypothesis, farms in the same regions tended to apply similar feeding regimes. Through discussion with farmers, we found that those in the same region (0.2–3.0 km apart) tended to learn farming practices from their neighbours. Comparing among feeding regime clusters, Cluster 1, which comprised all the NL SDFs, employed the most “best practice” feeding regimes; they fed cows with concentrates and roughages more times per day than average, cleaned feed troughs more often, supplied cows ad libitum good quality water, and mixed concentrate and roughage during feeding. In contrast, SDFs in feeding regime Cluster 2 (all SL SDFs) had the worst feeding regimes when they fed cow concentrate fewer times, used the same trough for both water and concentrate, and did not supplied cows ad libitum good quality water. These results suggest that extension programs need to be specific to each region. Specifically, NL SDFs should have separated water troughs instead of using the same troughs for both water and concentrates. They should also supply ad libitum water to cows, feed cows more frequently, and mix roughages and concentrates more properly when feeding cows. Smallholder dairy farms in SH and NH should mix roughage and concentrate more properly when feeding and clean water troughs more properly and often to ensure the cleanness of water. 

As expected, 29 out of 32 SDFs across regions did not weigh diet ingredients and none of the SDFs measure feed dry matter. This indicates that farmers did not formulate diets based on cows’ requirements. In addition, none of the SDFs mixed concentrates and roughages before feeding. This indicates that SDFs did not see the importance of mixing the feeds. Preparing total mixed rations based on cows’ requirements or at least mixing concentrate and roughage well when feeding can both improve performance and decrease the nutritional issues such as acidosis or laminitis [48,49]. For example, a study by Pilachai et al. [49] in Thai SDFs showed that the prevalence of subclinical laminitis was associated with feeding concentrate and roughage separately. Thus, further studies or extension programmes should aim to change these feeding practices. If possible, formulating diets into a total mixed ration (TMR) would be the best feeding practice. In Vietnam, Mai et al. [50] showed that feeding cows with TMR improved dry matter intake, milk yield, and milk quality compared to traditional feeding methods usually applied by SDFs. However, the same study also reported that the high cost of mixing wagons made it not economic for the SDF farmers [50]. Given this issue, farmers can at least do as one SDF in SH and four SDFs in NL did, which was to spread roughage into the feed trough first, then spread concentrate on the top of roughage, and then mix concentrates with roughage either by hand or using a rake.

The limited supply of good quality water for cows in all SL and SH SDFs is a serious problem. These farmers used the same trough for both concentrate and water and only supplied water for cows after they have eaten all the concentrates offered. A study by Lam et al. [15], also in SL, reported the same practice by SDF farmers, and so it appears that little has changed between then and the current study. This either reflects the conservatism of SDFs in these regions (SL and SH are among the regions with the longest dairy farming history in Vietnam) or the limitations of the extension systems in these regions [51]. Using the same trough for concentrate and water not only limits the water supplied to the cows but also promotes fermentation, making the water less drinkable [15]. In the SH region, although different troughs were used for water and concentrate, only two out of eight farms supplied water ad libitum for the cows. This result is consistent with previous surveys which reported that only 29% of SDFs in Hanoi [52] and 35% of SDFs in SL [15] supplying fresh water ad libitum for cows. When farmers were asked, in the current study, why they supplied limited water for cows, they answered that they wanted to ensure the high concentrations of milk fat and milk solid non-fat so that they can get a good price for their milk per litre. The limited supply of water not only raised welfare concerns but can also limit milk production. In tropical conditions, lactating dairy cows require 60–70 L of water per day for maintenance, plus an extra 4–5 L for each litre of milk production [53]. Insufficient water can also exacerbate the effects of high environmental heat load on the cows [54]. A study in the hot conditions of Pakistan showed that the provision of water ad libitum increased milk production by 1.5 kg/cow/day compared to only twice daily [55].

In the current study, apart from NL farms, all other regions fed cows concentrates twice per day, and roughage 2–3 times per day. These results are in line with the results of previous studies [15,16]. Again, this reflects the conservatism of the SDF farmers. Increasing feeding frequency, especially concentrate feeding frequency, is associated with increased feed intake, milk fat yield, and decreased severity of subacute ruminal acidosis in high producing cows [48,56,57]. When cows are fed more frequently, they will eat more evenly, which helps prevent rapid production of volatile fatty acids in the rumen caused by over-fermentation of starch [58,59].

### 4.2. Feeds and Diets

#### 4.2.1. Diversity and Quality of the Feeds

Feed types used by SDFs were diverse (19 types). As reported by other authors, concentrate pellets, fresh Napier grass, dry rice straw, fresh tropical grass, brewers’ grains, and the common roughages were the popular feeds used by SDFs [8,13,14,15,16]. However, the ratio of 0.39 kg of concentrate pellets fed per kg of FCM in current study was much lower than the 0.50 kg of concentrate pellets per kg of milk production reported by Cuong et al. [8]. Besides those feeds reported by previous authors, the current study found the use of many other feeds across regions. Corn silage, corn powders, whole soybean meal, and dried distillers’ grains, which are the common feeds for commercial dairy farms globally [9,60], were also used by Vietnamese SDFs. In addition, other industrialised feeds (PMR), industrial by-products (cassava residue and passion fruit pulp), agricultural by-products (fresh corn leaves, fresh rice straw, and rice hay), local feeds (rice grains with husk), silage (Napier grass silage), and some minerals and vitamin premixes were used. The diversity of the feeds used by SDFs could be the opportunity for formulating the least-cost or maximum-profit diets.

Compared with previous studies [8,13,14,15,16], the current study not only listed the feed types used by SDFs, but reported the diets that SDFs in each regions used for the cows and clustered the diets. Similar to feed types, the diets for the cows were diverse and made up nine diet clusters. Similar to feeding regimes, the SDFs in the same regions tended to feed cows similar diets (Diet clusters 3, 6, and 9), which again reflected habits of learning from neighbours of Vietnamese SDFs. The diversity and localisation of the diets implied that nutritionists or extensionists should formulate diets specific to each region. In addition, the feed companies who sell concentrate pellets should balance it according to the background ingredients specific to each region rather than produce one pellet formulation to fit all regions. In most cases, from the results of the current study, the commercial concentrate pellets should have a lower protein and a higher starch content to meet the actual nutrient requirements of the cows. In addition, Diet Cluster 6 was applied by many SDFs across regions, which implied the potential for SDFs to learn from other regions. This could the basis for the development of a more effective national extension program. 

Despite the diversity of the feed types found being used across regions, the quality of the roughages was a major issue. As reported by other authors, Napier grass and dry rice straw are especially fibrous with high concentrations of NDF (62.5–75.2% in Napier grass and 77.6% in dry rice straw), ADF (41.9–47.9% in Napier grass and 50.5% in dry rice straw), and ADL (5.3–11.5% in Napier grass and 5.4% in dry rice straw) [17,18,61,62]. However, unexpectedly, corn silage and fresh corn with cob did not showed much higher nutrient concentrations of NEL, CP, NFC or starch and did not show lower concentrations of NDF, ADF, and ADL than those of fresh Napier grass. In addition, the concentrations of NDF (56.3–70.1%), ADF (37.4–49.6%), and ADL (6.4–9.5%) of corn silages across regions in current study were higher than the normal means (± SD) of NDF (44.5 ± 4.9%), ADF (27.5 ± 3.9%), and ADL (4.0 ± 1.3%) concentrations in mature corn silage (32–38% DM) and much higher than the means (±SD) of NDF (44 ± 5.3%), ADF (28.1 ± 3.3%), and ADL (4.3 ± 1.0%) concentrations in mature corn silage (>40% DM) presented by NRC [9,60]. The high concentrations of NEL, CP, NFC and relatively low concentrations of NDF, ADF, and ADL are often of the reasons for higher nutrient digestibility of corn silage compared to other roughages such as Napier grass, making it one of the most suitable and most used roughages for dairy cows globally [9,60,63,64]. The high fibrous concentrations of the roughages are also a common reason for decreased feed intakes [9,60]. Thus, these results suggest that improving the quality of the roughages with a focus on reducing NDF, ADF, and ADL concentrations of not only Napier grass or rice straw but also corn silage and corn with cobs are crucial for improving the quality of smallholder dairy cow diets in Vietnam. The concentrations of ADF, NDF, and ADL often depend on the genetics of the forage as well as the stage of harvest [9]. The high fibrous concentrations of roughages in the current study suggested that SDFs famers might have not had suitable forage varieties or have harvested forages at too late.

The current study highlighted the wide variations of nutrient concentrations of the same feeds across regions, which suggested the importance of having different feed nutrient library for different regions. Through this study the nutritive values of 24 local feeds (11 feed types) were added into the feed library of PCDairy software for use by local nutritionists, extensionists and farmers. It serves as an important advance on what was previously available but could be further enhanced if the more feed samples per region could be analysed and at a variety of growth stages (e.g., season or harvesting age). 

#### 4.2.2. Imbalance and Inefficiency of the Diets

As predicted, the diets in all regions were insufficient in NEL concentration but excessive in CP, NDF, ADF, ADL and minerals. That SDF farmers did not weigh the feeds when feeding cows and the high fibre concentrations of all used roughages as discussed previously could be the reason for this dietary imbalance. Excess protein and minerals are normally eliminated through faeces and urine [9]. This both causes economic losses and environmental pollution [9]. The solutions to solve the diet imbalance issues in situation of Vietnamese SDFs could be either decrease CP and minerals concentrations in the diets or increasing NEL concentrations. The latter appears to be more beneficial as it could both balance the diets and increase milk production. As indicated by the results, the CP concentrations in SL, SH, and NH and minerals concentrations in all regions were even enough for a production of 25 kg of FCM. Thus, if the NEL concentration can be increased to be at least 1.59 MCal/kg DM, then a milk production of 25 kg FCM/cow/day could be expected.

The solution to increase NEL concentrations in the lactating cow diets can be increasing usages of starch and fat while decrease usages of high fibre roughage such as Napier grass or rice straw. In addition, improving starch and NFC but lowering fibre concentrations of corn silage are important. Currently, the NFC concentrations in the diets (24.9–29.3% DM) were all lower than a range of 30–42% DM suggested by Encyclopedia of Dairy Sciences [11] and PCDairy [12]. Starch concentration in diets (16.7–20.3% DM) were all lower than the range from 22–26% DM suggested by PCDairy [12]. Fat concentrations in diets (3.4–4.1% DM) were at the lower threshold of the range (3.0–6.0% DM) suggested by NRC [9] and PCDairy [12]. In contrast, the concentrations of NDF (43.9–47.4% DM), ADF (26.2–28.2% DM), and ADL (5.8–6.8% DM) in all regions were relatively high, and higher compared with the range 27–32% DM suggested for NDF and the range 19–21% DM suggested for ADF by Encyclopedia of Dairy Sciences [11] and PCDairy [12]. These indicate that there is still room for increasing starch and fat and decreasing the fibre concentrations of the diets whilst still ensuring rumen health. The risks when feeding higher starch and fat could be further reduced by taking more care to mix forages with concentrates at each feeding.

The feed efficiencies (0.99–1.19 kg FCM/kg DMI) in the current study were slightly lower than those values (1.07–1.14 kg FCM/kg DMI) in an experiment, which was also conducted in NH region of Vietnam [65], but much lower than those values in the studies conducted in the USA (1.39 and 1.72 kg FCM/kg DMI) [66,67]. 

Methane emission per kg FCM in the current study (16.5–21.4 g/kg FCM) was slightly lower than those (22.5–27.4 g CH_4_/kg FCM) in an experiment by Hiep et al. [68] which was conducted in Vietnam but much higher than those (13.1–14.8 CH_4_/kg energy corrected milk) in an experiment by Kolling et al. [69] in Brazil. Similarly, methane emission per kg of DMI in the current study (19.5–20.7 g CH_4_/kg DMI) was slightly higher than the values ranging 15.1–19.0 g CH_4_/kg DMI in an experiment by Hiep et al. [65], which was also conducted in diets for Vietnamese SDF cows, and higher to those (15.3–19.7 g CH_4_/kg DMI) in an experiment by Kolling et al. [69] in Brazil. 

Although the diet costs, calculated as USD/kg DMI, in SL and SH were both significantly cheaper than the diet cost in NH (*p* < 0.001, Table 7), the feed efficiency of the diet, calculated as kg FCM/kg DMI, in SL and SH were both significantly lower than that in NH (*p* = 0.016). As a result, the diet costs calculated for each kg of FCM were similar between all regions (0.39 USD/kg FCM, *p* = 0.846). Similarly, although methane emission calculated as g CH_4_/kg DMI was similar across regions (*p* = 0.064), the methane emission calculated as g CH_4_/kg FCM was highest in SL and lowest in NH (*p* = 0.013). This is because the feed efficiency of the diets in NH was higher than the feed efficiency of the diets in SL (1.10 vs 0.93 kg FCM/kg DMI, *p* = 0.014). This indicates that increasing milk production, which then increases feed efficiency, is a key to increase the cost-effectiveness of the diet and increase the environmental efficiency of milk production.

### 4.3. Limitations of the Study

The greatest difficulty in the current study was how to measure accurately feed intake, especially roughage intake. Concentrates were often only fed to lactating cows; thus, they can be measured easily. However, for roughage, farmers usually feed them to lactating cows and dry cows together. While lactating cows might consume more than dry cows, in the current study, intakes of roughage were calculated by dividing the total amount of roughage supplied to all cows including lactating cows and dry cows. Another issue was that three farmers fed roughage to cows at night-time when there was no observer to weigh that feed. We only asked farmers the next morning to bring the same amount as that they fed the cows at night and we weighed that amount. In addition, for a type of feed, while the nutrient composition feed refused might be different from that of the feed offered, the current study only used nutrient composition of the feed offered to calculate the nutrient intakes. All these factors might affect the accuracy of roughage intake measurements. 

## 5. Conclusions

SDFs within a given region tended to apply similar feeding regimes and feed cows similar diets. The most problematic region for each was SL. The feed types used by the SDFs were diverse and the nutrient concentrations of the same feed varied widely across regions. Therefore, the formulation of the diets and the extension programs to improve feeding practice for lactating cows should be specific to each region. Among regions, SL might require the most support. 

Feeding regimes, especially in SL, SH, and NH regions, could be improved supplying water ad libitum, increasing feeding frequency per day, and mixing feed before or during feeding. 

Lactating cow diets in all regions were excessive in protein, NDF, ADF, ADL, and minerals but insufficient in NEL, NFC, and starch. These imbalances may have caused the estimated sub-optimal feed efficiency and excessive methane emission of the cows fed these diets. The diets should be reformulated towards increased availability of starch and fat to replace high fibre roughages such as Napier grass or rice straw. Studies aimed at reducing the concentrations of NDF, ADF, and ADL concentrations in the most commonly used roughages by SDFs (Napier grass, corn silage, rice straw, and fresh corn with cob) are also required. Studies could include the selection of more suitable forage genotypes and the identification of optimal stage of harvest.

## Figures and Tables

**Table 1:** Feeding regime variables.

No	Variables	Data Collection Methods
	Quantitative variables	Count and/or ask farmers
1	Type of feeds offered to cows	Count
2	Roughage feeding times	Count
3	Concentrate feeding times	Count
4	Feed trough cleaning frequency (times/week)	Ask farmers
	Qualitative variables	Directly classify
5	Quantity of water supplied	*Ad libitum*, moderate, or insufficient
6	Quality of water supplied	Good, medium, or poor
7	Use same trough for water and concentrate	Yes or No
8	Use partial mixed ration	Yes or No
9	Weigh feeds before feeding	Yes or No
10	Measure feed dry matter	Yes or No
11	Feed concentrates and roughages separately	Yes or No
12	Mix concentrates and roughages during feeding	Yes or No

**Table 2:** Some background characteristics of lactating herds in each region.

Parameter	Region ^A^, Mean ± Standard Deviation				Reference
	SL	SH	NL	NH	
Lactating cows per farm	9 ± 5	6 ± 2	11± 2	18 ± 6	[22]
Lactation number	2.0 ± 0.4	2.5 ± 0.5	1.9 ± 0.7	2.5 ± 0.3	[22]
Per cent of first lactation cows	40 ± 26	20 ± 22	47 ± 23	35 ± 13	[22]
Per cent of second lactation cows	34 ± 32	32 ± 20	31± 12	26 ± 12	[22]
Days in milk, days	176 ± 82	172 ± 51	200 ± 58	177 ± 36	[22]
Body weight, kg	450 ± 29	496 ± 39	513 ± 49	535 ± 37	[22]
Breed categories					[23]
Pure Holstein	1 ± 4	37 ± 44	26 ± 40	98 ± 3	
7/8 Holstein:1/8 Zebu ^B^	59 ± 22	38 ± 37	63 ± 40	0 ± 0	
3/4 Holstein:1/4 Zebu	24 ± 21	24 ± 30	3 ± 4	0 ± 0	
Other breeds ^C^	16 ± 24	1 ± 4	8 ± 11	2 ± 3	
Space allowance, m^2^/cow	5.2 ± 1.1	7.8 ± 2.5	7.7 ± 2.8	14 ± 4.4	[24]

^A^ SL, South lowland; SH, South highland; NL, North lowland; NH, North highland. ^B^ Zebu cattle breeds in Vietnam include Red Sindhi, Vietnamese Yellow (Vang) cattle, and Lai Sind (Red Sindhi × Yellow). ^C^ Other breeds include 1/2 Holstein:1/2 Zebu, Brown Swiss, and Jersey.

**Table 3:** Comparisons of feeding regime for dairy cows between four main dairy regions.

Parameter	Region ^A^, Median or n ^B^				*p* ^C^	Overall ^D^
	SL	SH	NL	NH		
Quantitative variables (median)						Mean ± SEM
Type of feeds	4.5	5	4	5	0.845	4.6 ± 0.2
Roughage feeding times	3.5 ^a^	2.5 ^b^	4 ^a^	3 ^b^	<0.001	3.3 ± 0.3
Concentrate feeding times	2 ^b^	2 ^b^	4 ^a^	2 ^b^	<0.001	2.5 ± 0.5
Feed trough cleaning frequency (times/week)	10 ^ab^	7 ^b^	14 ^a^	7 ^b^	0.034	10.0 ± 2.0
Qualitative variables (n)						Frequency (%)
Supply water *ad libitum*	0 ^b^	2 ^ab^	7 ^a^	6 ^a^	<0.001	15 (47)
Same trough for water and concentrate	8 ^a^	0 ^b^	0 ^b^	0 ^b^	<0.001	8 (25)
Using partial mixed ration	0 ^b^	0 ^b^	0 ^b^	8 ^a^	<0.001	8 (25)
Water with visual contaminations	3	3	0	5	0.079	11 (34)
Weigh feeds before feeding	0	1	2	0	0.587	3 (9)
Measure feed dry matter	0	0	0	0	1.000	0 (00)
Feed concentrates and roughages separately	8 ^a^	0 ^b^	0 ^b^	7 ^a^	<0.001	15 (47)
Mix concentrates and roughages during feeding	0	1	4	0	0.034	5 (16)

^a,b^ Medians or frequencies with different superscript letters within a row differ significantly from each other, *p* < 0.05. ^A^ SL, South lowland; SH, South highland; NL, North lowland; NH, North highland. ^B^ Median for quantitative variables; n (number of farms out of eight farms) for qualitative variables. ^C^ *p*-values are given for either Kruskal–Wallis tests (superscript letters are given for post-hoc Wilcoxon rank sum test; *p* < 0.05) or Fisher’s exact tests (superscript letters are given for post-hoc Bonferroni-corrected pairwise Fisher’s exact test; *p* < 0.05). ^D^ Overall mean (SEM) of medians or overall frequency (percentage) of all farms.

**Table 4:** Feed ingredients used across smallholder dairy farms (n) and mean dry matter intake (kg DM/cow/day) of each feed ingredient for lactating cows in four major dairying regions across Vietnam.

Feed Intakes (kg DM/cow/day)	Region ^A^								*p* ^D^	Overall Mean ± SEM
	SL		SH		NL		NH			
	n ^B^	Mean ^C^	n	Mean	n	Mean	n	Mean		
Fresh Napier grass	6	2.6 ^ab^	8	5.0 ^a^	7	3.2 ^ab^	5	1.2 ^b^	0.009	3.0 ± 0.8
Fresh tropical grass	2	0.7	0	-	1	0.5	2	0.5	0.513	0.4 ± 0.1
Fresh corn with cob	0	-	2	1.1	2	0.7	1	0.2	0.430	0.5 ± 0.2
Fresh corn leaves	0	-	0	-	0	-	1	0.3	<0.001	0.1 ± 0.1
Corn silage	0	-	4	1.4 ^bc^	7	3.2 ^ab^	8	5.0 ^a^	<0.001	2.4 ± 1.1
Napier grass silage	0	-	0	-	1	0.2	0	-	<0.001	0.0 ± 0.0
Fresh rice straw	1	0.6	0	-	0	-	0	-	<0.001	0.2 ± 0.2
Dry rice straw	5	1.0	0	-	0	-	0	-	<0.001	0.3 ± 0.3
Rice hay	1	0.3	0	-	0	-	0	-	<0.001	0.1 ± 0.1
Partial mixed ration	0	-	0	-	0	-	8	2.0	<0.001	0.5 ± 0.5
Concentrate pellets	8	6.1	8	6.4	8	6.6	8	7.3	0.112	6.6 ± 0.2
Brewers’ grain	6	1.9 ^a^	3	0.6 ^b^	0	-	5	0.7 ^ab^	0.007	0.8 ± 0.4
Cassava residue	7	1.3	0	-	0	-	0	-	<0.001	0.3 ± 0.3
Corn powder	0	-	5	0.8 ^b^	8	1.6 ^a^	0	-	<0.001	0.6 ± 0.4
Whole soybean meal	0	-	1	0.1	2	0.1	0	-	0.272	0.1 ± 0.0
Passion fruit pulp	0	-	1	0.3	0	-	0	-	<0.001	0.1 ± 0.1
Sweet potato tuber	0	-	1	0.3	0	-	0	-	<0.001	0.1 ± 0.1
Dried distillers’ grain	0	-	2	0.2	0	-	0	-	<0.001	0.1 ± 0.1
Rice grain with husk	0	-	1	0.1	0	-	0	-	<0.001	0.0 ± 0.0

^A^ SL, South lowland; SH, South highland; NL, North lowland; NH, North highland. ^B^ n, number of farms using a given feed type out of eight farms per region. ^C^ Mean of eight farms; farms that did not use a feed were included as 0 kg DM/cow/day. ^D^ *p*-values were given for one-way ANOVA tests comparing means or superscript letters were given for post-hoc Tukey–Kramer test, *p* < 0.05. ^a–c^ Means with different superscript letters within a row differ significantly from each other, *p* < 0.05.

**Table 5:** Nutrient concentration of feeds commonly used for dairy cows in regions (on dry matters basis, otherwise stated) ^A^.

		Price	DM	NEL	TDN	CP	NDF	ADF	Fat	NFC	Starch	ADL	Ash	Ca	K	Mg	Na	P	S	Cu	Fe	Mn	Zn
No	Region—Feed Name	USD/ton	% AF	Mcal/kg	%	%	%	%	%	%	%	%	%	%	%	%	%	%	%	ppm	ppm	ppm	ppm
1	SL—Fresh Napier grass	38	19.8	1.08	58	15.8	65.2	41.9	2.2	10.0	0.2	5.3	6.8	0.27	3.19	0.17	0.02	0.32	0.14	7	234	56	55
2	SH—Fresh Napier grass	38	18.7	0.90	51	12.1	68.1	45.6	2.6	10.5	4.7	8.7	6.7	0.45	2.25	0.22	0.09	0.31	0.14	11	452	92	100
3	NL—Fresh Napier grass	38	19.1	0.77	49	10.1	72.5	47.9	2.7	7.9	1.0	10.1	6.8	0.51	1.74	0.36	0.09	0.34	0.18	8	605	91	33
4	NH—Fresh Napier grass	38	19.8	0.88	48	16.3	66.6	42.6	2.2	8.3	0.1	11.5	6.6	0.45	2.43	0.39	0.01	0.33	0.16	7	482	56	26
5	SH—Corn silage	104	23.8	1.28	61	10.4	56.3	37.4	3.0	25.1	17.8	6.4	5.2	0.22	1.23	0.18	0.90	0.23	0.13	7	735	84	27
6	NL—Corn silage	104	25.9	0.88	53	9.5	70.1	46.2	2.1	13.2	4.6	8.9	5.1	0.30	0.66	0.24	0.54	0.22	0.12	6	319	144	20
7	NH—Corn silage	104	26.3	0.90	52	13.0	69.0	49.6	2.0	10.8	0.7	9.5	5.2	0.41	1.73	0.50	0.01	0.23	0.14	6	317	33	25
8	SL—Fresh tropical grass	38	21.6	0.84	52	10.9	71.6	46.4	2.0	8.9	3.5	7.5	6.6	0.15	1.76	0.15	0.07	0.22	0.41	6	470	111	46
9	NL/NH—Fresh tropical grass	38	20.8	0.97	54	15.6	67.9	44.3	2.8	7.0	0.4	7.6	6.7	0.39	4.08	0.22	0.01	0.34	0.23	7	632	58	26
10	SH—Fresh corn with cob	75	25.0	1.19	61	12.4	61.4	38.2	2.3	18.7	15.1	5.1	5.2	0.27	1.60	0.13	0.04	0.22	0.13	6	688	114	37
11	NL—Fresh corn with cob	75	24.4	1.08	59	11.5	66.7	41.4	2.4	14.2	0.9	5.4	5.2	0.70	0.77	0.29	0.01	0.19	0.18	7	194	62	30
12	SL—Concentrate pellets	471	90.6	1.61	69	20.7	29.7	14.0	4.2	37.0	27.2	5.9	8.4	1.26	1.18	0.32	0.58	0.67	0.35	45	468	127	348
13	SH—Concentrate pellets	471	89.7	1.78	76	21.7	25.9	11.2	4.9	40.1	32.1	3.3	7.3	1.01	1.04	0.34	0.53	0.57	0.30	41	451	92	492
14	NL—Concentrate pellets	471	89.2	1.76	75	21.2	28.3	11.4	5.8	37.0	29.8	3.8	7.8	1.40	0.98	0.31	0.39	0.57	0.35	53	295	135	304
15	NH—Concentrate pellets	471	88.6	1.74	74	20.4	22.1	11.9	4.2	44.6	32.3	3.5	8.6	1.68	0.95	0.34	0.51	0.73	0.34	31	419	164	347
16	SL—Brewers’ grain	81	23.5	1.80	75	29.4	55.3	24.4	8.4	2.9	2.9	5.1	4.0	0.25	0.06	0.17	0.01	0.55	0.32	14	206	42	84
17	SH—Brewers’ grain	81	25.0	1.74	72	24.9	44.2	18.2	7.9	25.1	19.0	9.5	4.0	0.15	0.14	0.15	0.01	0.46	0.27	8	150	33	60
18	NH—Brewers’ grain	81	21.3	1.50	62	31.5	58.2	23.7	9.1	1.4	1.4	17.5	2.2	0.25	0.03	0.14	0.01	0.54	0.35	11	153	40	74
19	SH—Whole soybean meal	750	90.3	2.16	88	47.1	21.4	10.6	11.5	15.0	2.0	3.7	5.0	0.25	1.59	0.27	0.01	0.62	0.33	12	286	34	48
20	NL—Whole soybean meal	750	84.2	2.57	90	37.3	17.2	11.9	21.6	18.9	2.8	5.8	5.0	0.21	1.56	0.20	0.01	0.52	0.29	16	79	20	33
21	NH—Partial mixed ration	289	54.7	1.50	65	17.6	36.9	22.8	3.7	33.5	22.5	6.8	8.2	1.30	1.12	0.33	0.37	0.50	0.29	24	763	125	197
22	SL—Dry rice straw	173	84.2	0.68	53	7.2	77.6	50.5	1.4	6.8	0.5	5.4	7.0	0.37	1.96	0.16	0.15	0.13	0.18	4	532	287	35
23	SL—Cassava residue	54	19.8	1.63	72	2.5	31.2	25.6	0.1	63.2	48.6	4.8	3.0	0.50	0.23	0.10	0.02	0.02	0.02	1	190	39	12
24	SH—Passion fruit pulp	66	16.4	1.17	53	10.1	52.9	43.3	0.9	27.7	0.6	9.4	8.4	0.26	2.99	0.13	0.05	0.08	0.18	2	117	69	10

^A^ Feeds were sampled from SDFs and analysed at Dairy One Forage Laboratory. Abbreviations: SL, south lowland; SH, south highland; NL, north lowland; NH, north highland; USD, United States Dollar; AF, as fed; DM, dry matter; NEL, net energy for lactation; TDN, total digestible nutrients; CP, crude protein; NDF, neutral detergent fibre; ADF, acid detergent fibre; NFC, nonfibre carbohydrate; ADL, acid detergent lignin; Ca, calcium; P, phosphorus; K, potassium; Mg, magnesium; Na, sodium; S, sulphur; Cu, copper; Fe, iron; Mn, manganese; and Zn, zinc.

**Table 6:** Comparisons of average nutrient composition (DM basis; % unless otherwise noted) of the lactating cow diets between four dairy regions and between these regions with aspirational targets.

Nutrient ^A^ (DM Basis)	Actual Diets of Regions ^B^ (Mean)				*p* ^C^	Mean ± SEM	PCDairy Recommendations ^D^ (Mean ± SEM)		
	SL	SH	NL	NH			Recommen_1	Recommen_2	Max
Intake									
DMI, kg/cow/day	14.4 ^b^	16.4 ^ab^	16.2 ^ab^	17.3 ^a^	0.007	16.1 ± 0.6	--	--	--
DMI, % BW	3.2	3.3	3.2	3.3	0.861	3.2 ± 0.1	--	--	--
Concentration									
DM, % as fed	35.5 ^ab^	32.3 ^b^	36.6 ^ab^	39.2 ^a^	0.002	35.9 ± 1.4	--	--	--
NEL, MCal/kg	1.40	1.44	1.36	1.38	0.176	1.40 ± 0.02	1.50 ± 0.03	1.59 ± 0.02	--
TDN, %	64.7	63.7	65.4	63.4	0.481	64.3 ± 0.5	65.4 ± 2.0	69.9 ± 1.8	--
CP, %	17.1 ^a^	16.4 ^ab^	14.8 ^b^	17.5 ^a^	0.003	16.5 ± 0.6	13.8 ± 1.5	15.7 ± 0.5	--
NDF, %	47.4	43.9	46.9	44.9	0.192	45.8 ± 0.8	>28	>28	--
ADF, %	27.4	26.2	27.5	28.2	0.507	27.3 ± 0.4	>21	>21	--
Fat, %	3.6 ^ab^	3.9 ^ab^	4.1 ^a^	3.4 ^b^	0.026	3.8 ± 0.2	>3	>3	--
NFC, %	24.9	29.3	27.9	27.4	0.163	27.4 ± 0.9	--	--	--
Starch, %	16.7 ^b^	22.6 ^a^	20.3 ^ab^	16.8 ^b^	0.004	19.1 ± 1.4	--	--	--
ADL, %	5.8 ^ab^	5.5 ^b^	5.9 ^ab^	6.8 ^a^	0.012	6.0 ± 0.3	--	--	--
Ash, %	7.0	6.5	6.2	6.8	0.050	6.6 ± 0.2	--	--	--
Ca, %	0.71^b^	0.59 ^c^	0.78 ^b^	1.04 ^a^	<0.001	0.78 ± 0.10	0.49 ± 0.05	0.58 ± 0.03	2.0
P, %	0.44 ^b^	0.41 ^bc^	0.40 ^c^	0.49 ^a^	<0.001	0.44 ± 0.02	0.32 ± 0.03	0.37 ± 0.02	1.0
K, %	1.40	1.44	1.12	1.26	0.070	1.31 ± 0.07	0.9	0.9	3.0
Mg, %	0.25 ^b^	0.25 ^b^	0.28 ^b^	0.37 ^a^	<0.001	0.29 ± 0.03	0.20	0.20	0.5
Na, %	0.27	0.32	0.29	0.31	0.611	0.30 ± 0.01	0.18	0.18	1.6
S, %	0.26 ^a^	0.20 ^b^	0.23 ^ab^	0.26 ^a^	<0.001	0.24 ± 0.01	0.20	0.20	0.4
Cu, ppm	23 ^ab^	21 ^bc^	25 ^a^	18 ^c^	<0.001	22 ± 1	10	10	100
Fe, ppm	346 ^b^	437 ^a^	341 ^b^	402 ^ab^	0.001	381 ± 23	50	50	1000
Mn, ppm	101 ^a^	83 ^b^	108 ^a^	103 ^a^	0.003	99 ± 6	40	40	1000
Zn, ppm	174 ^b^	232 ^a^	137 ^c^	181 ^b^	<0.001	181 ± 19	40	40	500
Ca:P ratio	1.6 ^b^	1.5 ^b^	2.0 ^a^	2.1 ^a^	<0.001	1.8 ± 0.2	--	--	--

^A^ Abbreviations: DM, dry matter; NEL, net energy for lactation; TDN, total digestible nutrients; CP, crude protein; NDF, neutral detergent fibre; ADF, acid detergent fibre; NFC, nonfibre carbohydrate; Ca, calcium; P, phosphorus; K, potassium; Mg, magnesium; Na, sodium; S, sulphur; Cu, copper; Fe, iron; Mn, manganese; and Zn, zinc. ^D^ Recommended nutrient concentrations calculated internally by PCDairy for production of observed FCM (Recommen_1), for production of a target 25 kg FCM (3.5% fat) per day (Recommen_2), and the maximum concentrations of minerals (Max). ^B,C,a–c^ Other footnotes as in Table 4.

**Table 7:** Diet intakes (kg/cow/day), diet cost, prediction of milk yield (kg/cow/day), and methane emissions from the diet of cows in each region.

Parameter ^A^	Region ^B^, Mean				*p* ^C^	Mean ± SEM
	SL	SH	NL	NH		
Predicted milk production						
NEL-allowable FCM, kg/cow/day	16.5	20.6	17.6	19.8	0.049	18.6 ± 0.9
CP-allowable FCM, kg/cow/day	25.0 ^b^	27.6 ^ab^	23.9 ^b^	30.9 ^a^	<0.001	26.8 ± 1.6
CP-allowable FCM: NEL-allowable FCM	1.5	1.5	1.4	1.6	0.034	1.5 ± 0.1
Actual milk production and feed efficiency						
FCM, kg/cow/day	14.2 ^b^	16.2 ^b^	16.9 ^b^	20.4 ^a^	<0.001	16.9 ± 1.3
FCM, kg/kg DMI	0.99 ^b^	1.00 ^b^	1.04 ^ab^	1.19 ^a^	0.016	1.06 ± 0.05
Diet costs						
Diet cost, USD/cow/day	5.4 ^c^	6.4 ^bc^	6.5 ^b^	7.6 ^a^	<0.001	6.4 ± 0.4
Roughage cost, USD/cow/day	1.3 ^c^	2.0 ^bc^	2.3 ^b^	3.4 ^a^	<0.001	2.2 ± 0.5
Concentrate cost, USD/cow/day	4.2	4.4	4.2	4.0	0.765	4.2 ± 0.1
Diet cost, USD/kg DMI	0.37 ^b^	0.39 ^b^	0.40 ^ab^	0.44 ^a^	<0.001	0.40 ± 0.01
Diet cost, USD/kg FCM	0.39	0.39	0.39	0.37	0.846	0.39 ± 0.01
Predicted methane emissions						
CH_4,_ % of gross energy	5.54	5.31	5.46	5.24	0.049	5.39 ± 0.07
CH_4_, g/cow/day	297	324	329	336	0.056	321 ± 8
CH_4_, g/kg DMI	20.7	19.8	20.4	19.5	0.064	20.1 ± 0.3
CH_4_, g/kg FCM	21.4 ^a^	20.0 ^ab^	20.0 ^ab^	16.5 ^b^	0.013	19.5 ± 1.0

^A^ All results were calculated per cow per day. Abbreviations: DMI, dry matter intake; BW, body weight; FCM, fat corrected milk; NEL, net energy for lactation; CP, crude protein; USD, United States dollar. ^B,C,a–c^ Other footnotes as in Table 4.

## Data Availability

The data presented in this study are available on request from the corresponding author.

## Supplementary Materials

The following are available online at https://www.mdpi.com/2076-2615/11/3/729/s1, Figure S1: Measurements of feed offered, feed refused, and appearance of PCDairy software, Table S1: Most significant variables characterising each feeding regime clusters, Table S2: Most significant variables characterising each diet clusters.

## Appendix A

**Table A1:** Nutrient composition of feeds commonly used for dairy cows in regions (on dry matters basis, otherwise stated) ^A^

		Price	DM	NEL	TDN	CP	NDF	ADF	Fat	NFC	Starch	ADL	Ash	Ca	K	Mg	Na	P	S	Cu	Fe	Mn	Zn	
Feed Code ^B^	Region-Feed Name	USD/ton	% AF	Mcal/kg	%	%	%	%	%	%	%	%	%	%	%	%	%	%	%	ppm	ppm	ppm	ppm	Ref ^C^
551	NL-Napier grass silage	44	31.0	1.20	58.0	6.6	71.5	42.5	2.0	10.3	0.6	5.70	9.6	0.36	2.90	0.30	0.03	0.29	0.10	11	413	91	45	2
570	NL_SH-Corn powder	3462	88.0	2.00	88.7	9.3	9.5	3.2	4.3	75.3	70.0	0.09	1.6	0.03	0.37	0.14	0.03	0.30	0.12	3	35	6	27	1
581	SH-Rice grain with husk	200	89.0	1.70	81.4	9.0	11.0	9.0	1.8	72.2	66.0	0.90	6.0	0.07	0.53	0.14	0.07	0.36	0.05	3	57	20	71	1
582	SL-Rice hay	170	65.0	1.20	58.4	14.8	61.2	38.0	2.1	13.1	9.0	5.90	8.8	0.30	3.64	0.15	0.01	0.40	0.20	13	2	25	15	2
600	SL-Fresh rice straw	0	34.0	0.84	37.0	4.3	78.0	50.0	1.4	8.0	6.3	12.00	8.3	0.15	0.40	0.83	0.12	0.08	0.08	13	2	33	16	2
605	NH-Fresh corn leaves	58	27.4	1.44	65.0	11.4	63.6	31.8	2.0	21.8	3.5	3.70	2.2	0.63	0.17	0.35	0.01	0.15	0.22	11	104	256	69	2
607	SH-Dried distillers’ grain	330	89.0	2.21	82.0	29.5	34.2	13.6	11.1	11.0	9.3	4.30	5.4	0.16	1.03	0.33	0.24	0.79	0.40	6	123	21	62	1
608	SH-Sweet potato tuber	220	30.0	1.86	80.0	5.5	11.3	5.2	1.1	78.4	69.3	1.10	3.6	0.12	1.22	0.09	0.02	0.15	0.11	7	700	51	43	1

^A^ Abbreviations: SL, south lowland; SH, south highland; NL, north lowland; NH, north highland; USD, United States Dollar; AF, as fed; DM, dry matter; NEL, net energy for lactation; TDN, total digestible nutrients; CP, crude protein; NDF, neutral detergent fibre; ADF, acid detergent fibre; NFC, nonfibre carbohydrate; ADL, acid detergent lignin; Ca, calcium; P, phosphorus; K, potassium; Mg, magnesium; Na, sodium; S, sulphur; Cu, copper; Fe, iron; Mn, manganese; and Zn, zinc. ^B^ Feed code, code of feed in feed library of PCDairy-Vietnamese version. ^C^ Ref, references: 1, PCDairy—Vietnamese version [12]; 2, Feed nutritive value books [18].
