# Supplementary material for: Issues of Feeding Strategy for Lactating Cows in Vietnamese Smallholder Dairy Farms
Source: Animals (Basel). 2021 Mar 8;11(3):729. doi: 10.3390/ani11030729 (PMC7998530; doi:10.3390/ani11030729)
Supplement: Supplementary file 1 [file animals-11-00729-s001.pdf]

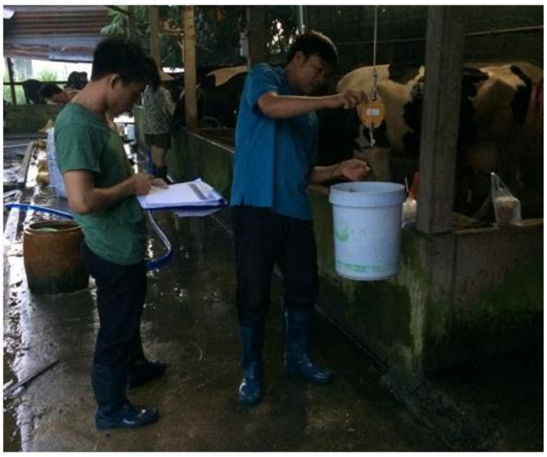

a) Weigh concentrate

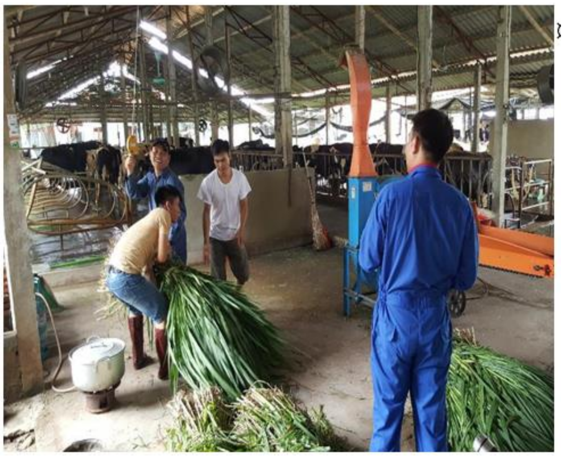

b) Weigh roughage

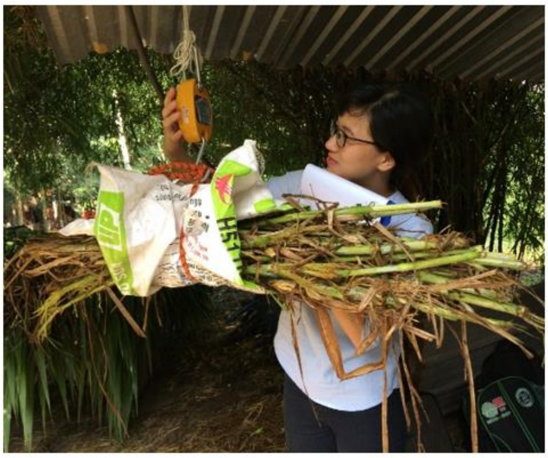

c) Weigh refusal

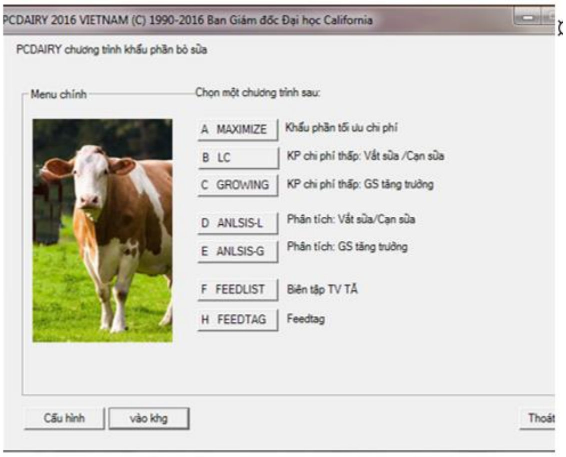

d) Run PCDairy

Figure S1. Measurements of feed offered, feed refused, and appearance of PCDairy software

Table S1. Most significant variables characterizing each feeding regime clusters

Cluster	Most significant variables [A]	Cluster mean (SD) or %	Overall mean (SD) or %	V.test	P [B]
C1	CoTim	3.7 (0.47)	2.5 (0.79)	5.14	<0.001
	FeTim	3.8 (0.42)	3.1 (0.74)	3.07	0.002
	FeCle	16.3 (5.72)	11.8 (6.44)	2.48	0.013
	MixDu=YesMixDu	55.6	15.6	3.42	0.001
	CoBeRo=NoCoBeRo	100	53.13	3.33	0.001
	WaQuTi=AdWaQuTi	88.9	46.9	2.86	0.004
	WaQuLi=GoWaQuLi	100	65.6	2.56	<0.001
	PMR=NoPMR	100	75.0	1.99	0.047
	SaCoWa=NoSaCoWa	100	75.0	1.99	0.047

	PMR=YesPMR	0.0	25.0	-1.99	0.047
	SaCoWa=YesSaCoWa	0.0	25.0	-1.99	0.047
	WaQuLi=MeWaQuLi	0.0	34.4	-2.56	0.010
	WaQuTi=MoWaQuTi	11.1	50.0	-2.65	0.008
	CoBeRo=YesCoBeRo	0.0	46.9	-3.33	0.001
	MixDu=NoMixDu	44.4	84.4	-3.42	0.001
C2	CoTim	2.0 (0.00)	2.5 (0.79)	-2.03	0.042
	SaCoWa= YesSaCoWa	100	25.0	5.34	<0.001
	CoBeRo=YesCoBeRo	100	46.9	3.43	0.001
	WaQuTi=MoWaQuTi	100	50.0	3.23	0.001
	WaQuTi=AdWaQuTi	0.0	46.9	-3.05	0.002
	CoBeRo=NoCoBeRo	0.0	53.1	-3.43	0.001
	SaCoWa=NoSaCoWa	0.0	75.0	-5.34	<0.001
C3	CoTim	2.1 (0.25)	2.5 (0.79)	-2.87	0.004
	FeTim	2.5 (0.50)	3.1 (0.74)	-4.18	<0.001
	PMR=YesPMR	53.3	25.0	3.43	0.001
	SaCoWa=NoSaCoWa	100	75.0	3.05	0.002
	MixDu=NoMixDu	100	84.4	2.16	0.031
	WaQuLi=MeWaQuLi	53.3	34.4	2.00	0.045
	WaQuLi=GoWaQuLi	46.7	65.6	-2.00	0.045
	MixDu=YesMixDu	0.0	15.6	-2.16	0.031
	SaCoWa= YesSaCoWa	0.0	25.0	-3.05	0.002
	PMR=NoPMR	46.7	75.0	-3.43	0.001

[A] Abbreviations: WaQuTi: if water supplied to cows ad libitum (AdWaQuTi), moderately (MoWaQuTi), or insufficiently (InWaQuTi); WaQuLi: if water quality is medium (MeWaQuLi) or good (GoWaQuLi); SaCoWa: if the same trough was used for both concentrate and water (YesSaCoWa) or not (NoSaCoWa); PMR: If partial mixed ration was used (YesPMR) or not (NoPMR); CoBeRo: if concentrates were fed before roughage (YesCoBeRo) of not (NoCoBeRo); MixDu: if concentrates and roughages were mixed during feeding time (YesMixDu) or not (NoMixDu); FeTim: times of feeding roughages per day; CoTim = times of feeding concentrates per day, FeCle: times of cleaning roughage trough per week. [B] P-values were from V.tests which compared the mean of each quantitative variable in each cluster with the mean of that variable in the whole dataset or compared the percentage of each category of each qualitative in each cluster with percentage of that category in the whole feeding regime dataset.

Table S2. Most significant variables characterizing each diet clusters

Cluster	Most significant variables	Cluster mean (SD), (kgDMI/cow/d)	Overall mean (SD), (kgDMI/cow/d)	V.test	P [A]
C1	Rice grain with husk	0.86 (0.00)	0.03 (0.15)	5.57	<0.001
	Sweet potato tuber	2.74 (0.00)	0.09 (0.48)	5.57	<0.001
C2	Dried distillers grain	0.63 (0.44)	0.06 (0.23)	4.47	<0.001
	Fresh corn with cobs	2.93 (2.56)	0.50 (1.32)	3.29	0.001
C3	Corn powder	1.72 (0.46)	0.60 (0.81)	4.83	<0.001
	Fresh Napier grass	4.50 (1.91)	3.02 (2.24)	2.31	0.021
	Whole soybean meal	0.17 (0.32)	0.05 (0.19)	2.22	0.027
C4	Fresh tropical grass	3.98 (0.02)	0.42 (1.07)	4.78	<0.001
C5	Passion fruit pulp	2.49 (0.00)	0.08 (0.43)	5.57	<0.001
C6	Partial mixed ration	1.76 (0.92)	0.50 (0.93)	4.75	<0.001
	Corn silage	5.42 (1.50)	2.40 (2.45)	4.30	<0.001
	Corn powder	0.07 (0.21)	0.60 (0.81)	-2.26	0.024
	Fresh Napier grass	1.29 (1.33)	3.02 (2.24)	-2.69	0.007
C7	Fresh rice straw	5.10 (0.00)	0.16 (0.89)	5.57	<0.001
	Cassava residue	2.26 (0.00)	0.33 (0.67)	2.88	0.004
C8	Rice hay	2.18 (0.00)	0.07 (0.38)	5.57	<0.001
C9	Dry rice straw	1.62 (0.40)	0.25 (0.61)	5.37	<0.001
	Cassava residue	1.17 (0.74)	0.33 (0.67)	3.03	0.002
	Brewer grain	1.85 (1.76)	0.78 (1.10)	2.33	0.020
	Corn silage	0.00 (0.00)	2.40 (2.45)	-2.35	0.019

[A] P values were from V.tests which compared the mean of each quantitative variable in each cluster with the mean of that variable in the whole the dataset or compared the percentage of each category of each qualitative in each cluster with percentage of that category in the whole Diet dataset.
